# Supplementary material for: Modeling glioblastoma heterogeneity as a dynamic network of cell states
Source: Mol Syst Biol. 2021 Sep 16;17(9):e10105. doi: 10.15252/msb.202010105 (PMC8444284; doi:10.15252/msb.202010105)
Supplement: Supplementary file 5 — Source Data for Figure 3 [file MSB-17-e10105-s001.zip › Figure3A_sourcedata/GSEA_3065/nosigdb_state3.GseaPreranked.1622718426028/NB_KI_CLUSTER3.html]

Details for gene set NB\_KI\_CLUSTER3[GSEA]

|  || Dataset | state3 |
| Phenotype | NoPhenotypeAvailable |
| Upregulated in class | na\_neg |
| GeneSet | NB\_KI\_CLUSTER3 |
| Enrichment Score (ES) | -0.49151123 |
| Normalized Enrichment Score (NES) | -1.8762196 |
| Nominal p-value | 0.0 |
| FDR q-value | 0.0025779922 |
| FWER p-Value | 0.03 |
Table: GSEA Results Summary

  

Fig 1: Enrichment plot: NB\_KI\_CLUSTER3      
 Profile of the Running ES Score & Positions of GeneSet Members on the Rank Ordered List

  

| PROBE | GENE SYMBOL | GENE\_TITLE | RANK IN GENE LIST | RANK METRIC SCORE | RUNNING ES | CORE ENRICHMENT || 1 | RTN3 |  |  | 50 | 0.435 | 0.0124 | No |
| 2 | HSP90AA1 |  |  | 60 | 0.402 | 0.0282 | No |
| 3 | HACD3 |  |  | 89 | 0.356 | 0.0399 | No |
| 4 | SGCB |  |  | 133 | 0.317 | 0.0482 | No |
| 5 | HLTF |  |  | 306 | 0.241 | 0.0383 | No |
| 6 | ATP6V0B |  |  | 319 | 0.238 | 0.0469 | No |
| 7 | FADS1 |  |  | 331 | 0.234 | 0.0555 | No |
| 8 | TRIM24 |  |  | 372 | 0.225 | 0.0602 | No |
| 9 | MMS22L |  |  | 645 | 0.179 | 0.0361 | No |
| 10 | HSPA8 |  |  | 678 | 0.175 | 0.0397 | No |
| 11 | MSH6 |  |  | 682 | 0.174 | 0.0466 | No |
| 12 | ZNF738 |  |  | 695 | 0.171 | 0.0524 | No |
| 13 | SEC61A2 |  |  | 739 | 0.165 | 0.0544 | No |
| 14 | FIGNL1 |  |  | 1070 | 0.133 | 0.0215 | No |
| 15 | ZNF519 |  |  | 1147 | 0.126 | 0.0180 | No |
| 16 | GPSM2 |  |  | 1200 | 0.121 | 0.0170 | No |
| 17 | POLE |  |  | 1305 | 0.115 | 0.0098 | No |
| 18 | RNF219 |  |  | 1334 | 0.113 | 0.0112 | No |
| 19 | SLC25A13 |  |  | 1342 | 0.112 | 0.0151 | No |
| 20 | CENPJ |  |  | 1459 | 0.105 | 0.0060 | No |
| 21 | TOPBP1 |  |  | 1474 | 0.105 | 0.0088 | No |
| 22 | VPS45 |  |  | 1507 | 0.103 | 0.0094 | No |
| 23 | WDR54 |  |  | 1513 | 0.102 | 0.0131 | No |
| 24 | FSD1L |  |  | 1745 | 0.089 | -0.0101 | No |
| 25 | SCCPDH |  |  | 1808 | 0.086 | -0.0137 | No |
| 26 | RRP7A |  |  | 1820 | 0.086 | -0.0114 | No |
| 27 | CPSF3 |  |  | 1853 | 0.084 | -0.0116 | No |
| 28 | CEP78 |  |  | 1933 | 0.079 | -0.0175 | No |
| 29 | ACTR6 |  |  | 1948 | 0.078 | -0.0158 | No |
| 30 | EXOSC8 |  |  | 1955 | 0.078 | -0.0132 | No |
| 31 | VKORC1L1 |  |  | 2101 | 0.072 | -0.0271 | No |
| 32 | ZNF620 |  |  | 2229 | 0.067 | -0.0391 | No |
| 33 | KLHL42 |  |  | 2260 | 0.066 | -0.0398 | No |
| 34 | METTL2A |  |  | 2383 | 0.061 | -0.0514 | No |
| 35 | KLHL23 |  |  | 2474 | 0.058 | -0.0595 | No |
| 36 | SYNC |  |  | 2631 | 0.052 | -0.0754 | No |
| 37 | NSUN5 |  |  | 2659 | 0.051 | -0.0764 | No |
| 38 | ADAT1 |  |  | 2686 | 0.050 | -0.0773 | No |
| 39 | HSPD1 |  |  | 2700 | 0.050 | -0.0768 | No |
| 40 | BAZ1B |  |  | 2755 | 0.049 | -0.0810 | No |
| 41 | DLD |  |  | 2811 | 0.047 | -0.0855 | No |
| 42 | KNOP1 |  |  | 2880 | 0.045 | -0.0915 | No |
| 43 | LDHB |  |  | 3031 | 0.040 | -0.1073 | No |
| 44 | PARP2 |  |  | 3073 | 0.039 | -0.1104 | No |
| 45 | MFAP1 |  |  | 3091 | 0.039 | -0.1107 | No |
| 46 | PRMT1 |  |  | 3103 | 0.038 | -0.1104 | No |
| 47 | HSF2 |  |  | 3140 | 0.038 | -0.1130 | No |
| 48 | SNX4 |  |  | 3250 | 0.035 | -0.1243 | No |
| 49 | CCP110 |  |  | 3323 | 0.033 | -0.1313 | No |
| 50 | PSMC4 |  |  | 3328 | 0.032 | -0.1304 | No |
| 51 | HPF1 |  |  | 3472 | 0.029 | -0.1458 | No |
| 52 | CTDSPL2 |  |  | 3487 | 0.028 | -0.1463 | No |
| 53 | COMMD2 |  |  | 3499 | 0.028 | -0.1464 | No |
| 54 | TRAP1 |  |  | 3617 | 0.025 | -0.1589 | No |
| 55 | NDUFA4 |  |  | 3699 | 0.023 | -0.1674 | No |
| 56 | SFXN1 |  |  | 3852 | 0.019 | -0.1843 | No |
| 57 | GOT1 |  |  | 3908 | 0.017 | -0.1900 | No |
| 58 | FANCL |  |  | 3970 | 0.016 | -0.1964 | No |
| 59 | ASNS |  |  | 4228 | 0.009 | -0.2260 | No |
| 60 | UTP18 |  |  | 4406 | 0.004 | -0.2464 | No |
| 61 | DNAJC2 |  |  | 4461 | 0.002 | -0.2526 | No |
| 62 | DDX1 |  |  | 4490 | 0.001 | -0.2558 | No |
| 63 | PPP1R35 |  |  | 4690 | -0.003 | -0.2788 | No |
| 64 | NLN |  |  | 4802 | -0.006 | -0.2915 | No |
| 65 | UNG |  |  | 4979 | -0.010 | -0.3115 | No |
| 66 | SSBP2 |  |  | 5047 | -0.012 | -0.3188 | No |
| 67 | FXR2 |  |  | 5229 | -0.016 | -0.3392 | No |
| 68 | HIRIP3 |  |  | 5291 | -0.018 | -0.3455 | No |
| 69 | PRR3 |  |  | 5563 | -0.026 | -0.3760 | No |
| 70 | FLAD1 |  |  | 5715 | -0.030 | -0.3923 | No |
| 71 | CTCF |  |  | 5766 | -0.032 | -0.3968 | No |
| 72 | NEK1 |  |  | 5800 | -0.033 | -0.3992 | No |
| 73 | EIF4E |  |  | 5835 | -0.034 | -0.4018 | No |
| 74 | PSMB4 |  |  | 5904 | -0.036 | -0.4082 | No |
| 75 | VPS72 |  |  | 5989 | -0.039 | -0.4163 | No |
| 76 | MRPL44 |  |  | 6111 | -0.043 | -0.4286 | No |
| 77 | WDR18 |  |  | 6144 | -0.044 | -0.4305 | No |
| 78 | SPOP |  |  | 6228 | -0.047 | -0.4381 | No |
| 79 | FKBP4 |  |  | 6339 | -0.051 | -0.4488 | No |
| 80 | COPS7B |  |  | 6442 | -0.054 | -0.4584 | No |
| 81 | METAP2 |  |  | 6486 | -0.056 | -0.4611 | No |
| 82 | NUFIP1 |  |  | 6555 | -0.059 | -0.4665 | No |
| 83 | MRPL10 |  |  | 6624 | -0.062 | -0.4718 | No |
| 84 | DUS1L |  |  | 6628 | -0.062 | -0.4696 | No |
| 85 | SLC25A17 |  |  | 6631 | -0.063 | -0.4672 | No |
| 86 | HDGFL2 |  |  | 6687 | -0.065 | -0.4708 | No |
| 87 | ENOPH1 |  |  | 6746 | -0.068 | -0.4747 | No |
| 88 | NDUFAF6 |  |  | 6754 | -0.069 | -0.4727 | No |
| 89 | SINHCAF |  |  | 6769 | -0.070 | -0.4714 | No |
| 90 | SSX2IP |  |  | 6781 | -0.070 | -0.4697 | No |
| 91 | JPT2 |  |  | 6804 | -0.071 | -0.4693 | No |
| 92 | GPS1 |  |  | 6836 | -0.072 | -0.4699 | No |
| 93 | INPP5F |  |  | 6837 | -0.072 | -0.4668 | No |
| 94 | PRPSAP2 |  |  | 6890 | -0.075 | -0.4697 | No |
| 95 | CASP3 |  |  | 6969 | -0.079 | -0.4755 | No |
| 96 | GARS |  |  | 6979 | -0.079 | -0.4732 | No |
| 97 | SUCLG1 |  |  | 7007 | -0.082 | -0.4729 | No |
| 98 | GIHCG |  |  | 7022 | -0.082 | -0.4711 | No |
| 99 | GPN1 |  |  | 7075 | -0.085 | -0.4736 | No |
| 100 | UBB |  |  | 7097 | -0.087 | -0.4724 | No |
| 101 | MAGOHB |  |  | 7103 | -0.088 | -0.4693 | No |
| 102 | DBNL |  |  | 7145 | -0.090 | -0.4703 | No |
| 103 | SH3GLB2 |  |  | 7189 | -0.093 | -0.4714 | No |
| 104 | ENO1 |  |  | 7257 | -0.097 | -0.4751 | No |
| 105 | RFK |  |  | 7315 | -0.102 | -0.4774 | No |
| 106 | SNRPC |  |  | 7387 | -0.107 | -0.4812 | No |
| 107 | ACYP1 |  |  | 7427 | -0.110 | -0.4811 | No |
| 108 | NELFE |  |  | 7517 | -0.119 | -0.4865 | Yes |
| 109 | MANBAL |  |  | 7561 | -0.123 | -0.4863 | Yes |
| 110 | DCAKD |  |  | 7566 | -0.123 | -0.4816 | Yes |
| 111 | CDKN2C |  |  | 7609 | -0.128 | -0.4811 | Yes |
| 112 | LCMT1 |  |  | 7680 | -0.137 | -0.4835 | Yes |
| 113 | CISD1 |  |  | 7700 | -0.139 | -0.4799 | Yes |
| 114 | CACYBP |  |  | 7708 | -0.141 | -0.4748 | Yes |
| 115 | SEPHS2 |  |  | 7716 | -0.141 | -0.4697 | Yes |
| 116 | EIF3I |  |  | 7721 | -0.142 | -0.4642 | Yes |
| 117 | CARHSP1 |  |  | 7812 | -0.154 | -0.4682 | Yes |
| 118 | CCDC25 |  |  | 7842 | -0.157 | -0.4650 | Yes |
| 119 | ACOT7 |  |  | 7857 | -0.159 | -0.4600 | Yes |
| 120 | FAM104A |  |  | 7957 | -0.178 | -0.4640 | Yes |
| 121 | TUBG1 |  |  | 7966 | -0.179 | -0.4574 | Yes |
| 122 | EIF5A |  |  | 8048 | -0.193 | -0.4588 | Yes |
| 123 | PGAM1 |  |  | 8093 | -0.203 | -0.4554 | Yes |
| 124 | TAF9 |  |  | 8142 | -0.214 | -0.4520 | Yes |
| 125 | MRPL17 |  |  | 8154 | -0.216 | -0.4442 | Yes |
| 126 | CCDC167 |  |  | 8164 | -0.219 | -0.4361 | Yes |
| 127 | IFT22 |  |  | 8172 | -0.220 | -0.4277 | Yes |
| 128 | SET |  |  | 8175 | -0.221 | -0.4186 | Yes |
| 129 | ARHGDIA |  |  | 8218 | -0.233 | -0.4137 | Yes |
| 130 | SLC25A4 |  |  | 8291 | -0.256 | -0.4114 | Yes |
| 131 | AKR1B1 |  |  | 8304 | -0.259 | -0.4019 | Yes |
| 132 | EIF4EBP1 |  |  | 8316 | -0.262 | -0.3922 | Yes |
| 133 | SLC25A39 |  |  | 8335 | -0.266 | -0.3831 | Yes |
| 134 | ARPC5L |  |  | 8342 | -0.270 | -0.3725 | Yes |
| 135 | COA3 |  |  | 8345 | -0.272 | -0.3613 | Yes |
| 136 | NT5DC2 |  |  | 8363 | -0.279 | -0.3516 | Yes |
| 137 | UBE2M |  |  | 8372 | -0.281 | -0.3408 | Yes |
| 138 | ACTG1 |  |  | 8389 | -0.289 | -0.3305 | Yes |
| 139 | HRAS |  |  | 8396 | -0.293 | -0.3189 | Yes |
| 140 | SRP14 |  |  | 8397 | -0.293 | -0.3066 | Yes |
| 141 | CYCS |  |  | 8405 | -0.297 | -0.2950 | Yes |
| 142 | PKM |  |  | 8420 | -0.301 | -0.2840 | Yes |
| 143 | HMGN1 |  |  | 8432 | -0.308 | -0.2723 | Yes |
| 144 | COX5B |  |  | 8448 | -0.314 | -0.2609 | Yes |
| 145 | MRPL33 |  |  | 8504 | -0.342 | -0.2530 | Yes |
| 146 | HMGA1 |  |  | 8519 | -0.346 | -0.2401 | Yes |
| 147 | CAMTA1 |  |  | 8540 | -0.357 | -0.2274 | Yes |
| 148 | COPRS |  |  | 8556 | -0.368 | -0.2138 | Yes |
| 149 | NDUFAF8 |  |  | 8561 | -0.373 | -0.1986 | Yes |
| 150 | ODC1 |  |  | 8576 | -0.385 | -0.1841 | Yes |
| 151 | ANAPC11 |  |  | 8633 | -0.439 | -0.1722 | Yes |
| 152 | RPL37A |  |  | 8636 | -0.440 | -0.1539 | Yes |
| 153 | YBX1 |  |  | 8659 | -0.467 | -0.1369 | Yes |
| 154 | TXN |  |  | 8662 | -0.467 | -0.1176 | Yes |
| 155 | RAC1 |  |  | 8673 | -0.484 | -0.0984 | Yes |
| 156 | PPP1R14B |  |  | 8674 | -0.484 | -0.0781 | Yes |
| 157 | ARPC1A |  |  | 8680 | -0.493 | -0.0580 | Yes |
| 158 | CFL1 |  |  | 8698 | -0.516 | -0.0384 | Yes |
| 159 | FABP5 |  |  | 8699 | -0.518 | -0.0167 | Yes |
| 160 | TPI1 |  |  | 8709 | -0.538 | 0.0049 | Yes |
Table: GSEA details [plain text format]

  

Fig 2: NB\_KI\_CLUSTER3: Random ES distribution      
 Gene set null distribution of ES for **NB\_KI\_CLUSTER3**
